# Single-platform, volumetric, CD45-assisted pan-leucogating flow cytometry for CD4 T lymphocytes monitoring of HIV infection according to the WHO recommendations for resource-constrained settings

**DOI:** 10.1186/1756-0500-6-169

**Published:** 2013-04-30

**Authors:** Donato Koyalta, Mohammad-Ali Jenabian, Ngamasra Nadjiouroum, Barou Djouater, Noël Djemadji-Oudjeil, Angélique Ndjoyi-Mbiguino, Laurent Bélec

**Affiliations:** 1 Ministère de la Santé Publique, N’Djamena BP 407, Chad; 2 Chronic Viral Illness Service and Research Institute of the McGill University Health Centre, Montreal, Canada; 3 Hôpital de la Liberté, N’Djamena, Chad; 4 Conseil National de Lutte contre le SIDA, Primature, N’Djamena, Chad; 5 Organisation Mondiale de la Santé, Représentation du Tchad, N’Djamena, Chad; 6 Laboratoire National de Référence des Maladies Sexuellement Transmissibles et du SIDA, Département de Microbiologie, Faculté de Médecine de Libreville, Université des Sciences de la Santé, Libreville, Gabon; 7 Laboratoire de virologie, hôpital Européen Georges Pompidou, and Faculté de Médecine Paris Descartes, Université Paris Descartes (Paris V), Sorbonne Paris Cité, Paris, France

**Keywords:** Flow cytometry, CD4 T cell count, Pan-leucogating, CD45, Sub-Saharan Africa, Chad

## Abstract

**Background:**

Validation of new affordable CD4 T cell measurement technologies is crucial specifically in resource-poor countries for antiretroviral treatment eligibility and immunologic CD4 monitoring of HIV-infected patients.

**Methods:**

The absolute and percentage CD4 T cell counts of 258 HIV-1-infected blood samples (182 adults and 76 children), living in N’Djamena, Chad, were performed by single-platform, volumetric, CD45-assisted pan-leucogating Auto40 flow cytometer (Apogee Flow Systems Ltd, Hemel Hempstead, UK) comparing to the FACSCalibur flow cytometer as a reference method.

**Results:**

Absolute and percentage CD4 T cell counts obtained by Auto40 and FACSCalibur of 258 HIV-1-infected blood samples were highly correlated (r = 0.99 and r = 0.96, respectively). The mean absolute bias and percent bias between Apogee Auto40 and FACSCalibur absolute CD4 T cell counts, were −9.4 cells/μl with limits of agreement from −15 to 93 cells/μl, and +2.0% with limits of agreement from −0.9 to 4.9%, respectively. The mean of absolute bias and percent bias between Apogee Auto40 and FACSCalibur of CD4 percentage results were +0.4% (95% CI: -0.02 – 0.86) with limits of agreement from −2.4 to 0.3%, and +3.0% with limits of agreement from −6.6 to 0.6%, respectively. The Auto40 counting allowed to identify the majority of adults with CD4 T cells below 200 cells/μl (sensitivity: 89%; specificity: 99%) or below 350 cells/μl (sensitivity: 94%; specificity:98%); and of children below 750 cells/μl (sensitivity: 99%; specificity: 96%) or below 25% CD4+ (sensitivity: 94%; specificity: 98%).

**Conclusion:**

The Auto40 analyzer is an alternative flow cytometer for CD4 T lymphocyte enumeration to be used in routine for immunological monitoring according to the current WHO recommendations in HIV-infected adults as well as children living in resource-constrained settings like Chad.

## Background

The HIV epidemic remains a major global public health challenge with a total of 33.4 million people living with HIV worldwide [1]. The past decade has witnessed a remarkable global effort to improve access to HIV antiretroviral therapy (ART). The current guidelines of the World Health Organization (WHO) for scaling up of ART in adults and children living in resource-limited settings [2,3], emphasize the necessity of laboratory monitoring, which is initially based on immunological assessment by the enumeration of CD4 T lymphocytes, mainly to start ART and monitor patients on ART. In addition, HIV-1 RNA load is now recommended in order to monitor treatment efficacy and early therapeutic failure in patients on ART as well as to monitor therapeutic switch [4,5].

CD4 T lymphocytes monitoring typically relies on complex flow cytometry equipment which requires infrastructure and technical skills which are commonly unavailable at rural and remote clinics [6]. New point-of-care (POC) CD4 technologies enable testing to be decentralized to these sites and for test results to be provided during the course of the patient visit [7]. Recent studies have demonstrated that POC CD4 T lymphocytes monitoring can significantly improve rates of ART initiation and reduce patient loss-to-follow-up, which is often high before treatment initiation [1,8,9]. Immediate access to CD4 T cells results may also enable more rapid initiation of prophylactic treatment for opportunistic infections as well as chemotherapy for prevention of mother-to-child transmission at sites where CD4 T lymphocytes levels define the prophylactic drug regimen [10,11]. Affordable CD4 T cell counting has gradually become possible by using simple, compact and robust low-cost new generation of POC flow cytometers operating as single-platform volumetric instruments without the use of expensive microbeads [7,12-14]. Introduced in 2005, the recently developed Auto40 flow cytometer (Apogee Flow Systems Ltd, Hemel Hempstead, UK; http://www.Apogeeflow.com) was originally designed for military applications [15]. The Auto40 assay is based on no-lyse procedure [16], which avoids the red blood cell lysis step, thereby reducing assay variability due to changes in assay conditions (time and temperature of incubation) as well as differences in the susceptibility of cells to the lysis reagents [17]. The Auto40 analyzer uses a volumetric syringe moved by a stepper motor that draws and delivers a known sample volume. Therefore, its absolute volumetric counting allows the direct determination of the number of cells per unit of sample volume without the need for reference material such as microbeads [13,18].

Due to the stability of its optical bench, the Auto40 may be used on peripheral stationary as well as mobile flow cytometry unit [15]. The Auto40 analyzer was initially intended for CD4 T cell enumeration based on primary CD4 gating, and has been validated for measurement of absolute CD4 T cell count by reference to the FACSCount system [15]. The analyzer has been recently updated for CD4 T cells measurement within 30 minutes, using pan-leucogating protocol with primary CD45 gating followed by secondary CD4 gating, that allows results to be obtained in a single measurement of the CD4 T cell count expressed both in absolute number and in percentage [19,20], and therefore to address the current WHO recommendations for CD4 T cell measurements in children less than 5 years [2]. The initial version of Auto40 analyzer based on primary CD4 gating for CD4 T cell enumeration in absolute number has been evaluated positively in [15]; and the current version is based on CD45-assisted panleucogating for CD4 T cell enumeration in absolute number as well as in percentage in Cameroon [19,20].

The WHO strongly recommends scientific publications of effective validation of newly introduced affordable CD4 T cell measurement technologies carried out in the field by several laboratories of different resource-poor countries, and independently of manufacturers [6,21]. Therefore, the objective of the present study was to perform a novel and independent evaluation of the biological performances of the Auto40 analyzer in the HIV dedicated reference laboratory of the hôpital Militaire d’Instruction, located in N’Djamena, the capital city of Republic of Chad.

## Methods

### Clinical specimens and processing

Tripotassium ethylenediamine tetraacetate (K3-EDTA)-blood samples obtained by venipuncture in Vacutainer tubes (Becton Dickinson, Franklin Lakes, NJ, USA) were consecutively received in April 2012 (during the dry season) for routine biological monitoring at the reference laboratory of the hôpital de la Liberté, N’Djamena, an institutional reference laboratory devoted to HIV screening and monitoring. Two aliquots of one sample were kept at ambient temperature. No extra specimens were required. All blood samples were unlinked to identifiers. Each aliquot was first subjected to immediate measurement by flow cytometry reference analyzer, at ambient temperature at the hôpital de la Liberté, N’Djamena, and the second aliquot was sent within 4 hours to the hôpital Militaire D’Instruction, N’Djamena, for CD4 T cell count by Auto40 flow cytometer.

The study was approved by the Chad Ministry of Public Health. Written informed consents from all subjects were obtained before study initiation. For children samples, legal children’s guardians or parents signed an informed consent.

### CD4 T cell quantification and validation of the Auto40 flow cytometer comparing with the FACSCalibur reference flow cytometry method

CD4 T cell quantification was performed in parallel on 2 different flow cytometers: (1) the FACSCalibur [Becton Dickinson Immuno-cytometry System (BDIS), San Jose, CA, USA], a dedicated clinical reference flow cytometer for CD4 T cell counting installed at the hôpital de la Liberté, N’Djamena, using Multitest reagent (BDIS) and MultiSet V2.2 software (BDIS) for calculating the absolute and percentage values of CD4 T cells according to the manufacturer instructions, as previously described [22,23]; and (2) the Auto40 flow cytometer (Apogee Flow Systems Ltd) equipped with a green laser at 532 nm, a side scatter detector, two fluorescence channels and means for direct volumetric counting, without requiring a red blood cell lysis step.

**Figure 1:**
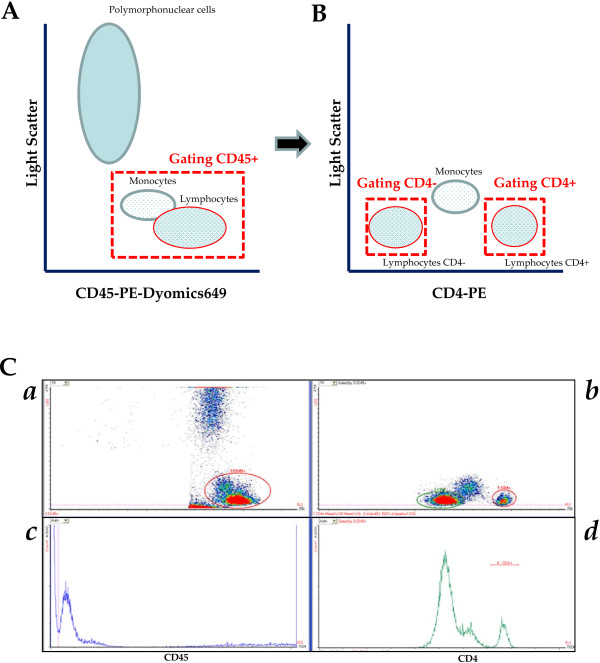
**Gating strategy for volumetric, CD45-assisted panleucogating Auto40 flow cytometer (Apogee Flow Systems Ltd, Hemel Hempstead, UK), as depicted from typical figure provided by “Auto-Lymphocyte” software (Apogee Flow Systems Ltd) for the counting of CD4 T lymphocytes: A**. CD45-PE-Dyomics649 *x* axis-light scatter dot plot scattergram; the primary CD45 gating differentiates clearly the CD45-positive lymphocytes and monocyte populations, and the population of polymorphonuclear cells; **B**. CD4-PE *x* axis-light scatter dot plot scattergram; double secondary CD4 gatings allow counting independently the population of CD4-negative lymphocyte and CD4-positive lymphocytes; **C**. Representative figure depicted by the software “Auto-Lymphocyte” in one blood sample taken as example: (***a***) CD45 (FL2)xside scatter (LS2) dot plot scattergram; (***b***) CD4 (FL1)xside scatter (LS2) dot plot scattergram; (***c***) Distribution of bright CD45 events during blood sample counting; (***d***) Distribution of bright CD4 events within the primary CD45 gating scattergram, during blood sample counting.

Direct volumetric CD4 T cell measurements were performed on the Auto40 using phycoerythrin (PE)-conjugated anti-CD4 and PE-Dyomics649-conjugated anti-CD45 monoclonal antibodies (Apogee Flow Systems Ltd). The Auto40 analytical procedure avoids the need for a wash step. Briefly, 50 μl of whole EDTA-blood was added into polypropylene test tubes containing pre-dispensed, stabilized monoclonal antibodies. After 25 minutes of incubation at room temperature in the dark, the blood samples were diluted 1:10 in phosphate buffered saline. The no-lyse-no-wash stained samples were run on the Auto40 flow cytometer, and CD4 T cell count was obtained in absolute number and in percentage. Analysis on the Auto40 flow cytometer was automatically performed by the built-in software “Auto-lymphocyte” (Apogee Flow Systems Ltd), with the possibility of controlling and assessing the quality of the data analysis. CD45-positive lymphocytes and monocytes were identified by primary gating on bright CD45 fluorescent cells in a CD45xside scatter dot plot scatter gram (Figure 1A). The CD45 fluorescent polymorphonuclear cells were excluded from the gating according to their high nuclear density. Within the CD45-positive cells, CD4-positive and CD4-negative lymphocytes were identified by secondary gating in a CD4xside scatter dot plot scatter gram (Figure 1B). CD4-positive T lymphocytes are easily separated for monocytes and CD4-negative lymphocytes, and counted independently. The CD4 T lymphocytes count in absolute number corresponds to the CD4-positive secondary CD4 gating cell populations. The CD4 T lymphocytes count in percentage corresponds to the ratio of CD4-positive lymphocytes count in absolute number to the total CD4-positive and CD4-negative lymphocytes counts, corresponding to the sum of both secondary CD4 gating. The sample reading takes around 2 minutes. Aside from simplifying sample preparation, the software “Auto-lymphocyte” allows automatic reading, and greatly facilitates manipulations by laboratory technicians. The whole procedure is fast and only needs 30 minutes to be completed. According to the manufacturer, the Auto40 flow cytometer has the theoritical capacity of 150 CD4 T cells enumerations per day.

To ensure quality control of the flow cytometric immune-phenotyping method with regard to the performance of the personnel and the instrument, the same lots of reagents were used throughout the study, and all sample preparations and flow cytometric analyses were performed by the same operator for each instrument, as previously reported [24]. Adequate training on the use of reverse pipetting technique and electronic pipette was also provided for each operator. Furthermore, the FACSCalibur photomultiplier tube voltage, sensitivity, and fluorescent compensation settings were optimized prior to sample acquisition. In addition, weekly calibration and internal quality control of the FACSCalibur were performed by using Calibrite beads (BDIS) and FACSComp software (BDIS). Quality control on the Auto40 flow cytometer was primarily assessed by the use of a calibrated bead sample (Apogee cat # 1444 for Auto40-green, Apogee Flow Systems Ltd) at the beginning and end of each session, and on the use of stabilized blood reference samples (Cytofix CD4, CaltagMedsystems Ltd, UK) as an additional external control.

### Statistical analyses

The Method Validator software version 1.1.9.0. (Philippe Marquis, France) and the SAS-PC software (version 8.2, SAS Institute, Cary, North Carolina, USA) were used for statistical analyses. First, correlations between the absolute CD4 T cell counts obtained by the reference FACSCalibur and the Auto40 were established by the Passing-Bablok nonparametric method [25]. Secondly, the agreement between FACSCalibur and Auto40 was depicted by difference plots as proposed by Bland and Altman [25-27] and Pollock et al. [28]. The Bland-Altman and Pollock analyses were carried out to calculate the mean of absolute and relative bias and limits of agreement, respectively, corresponding to the 95% confidence intervals [± 1.96 × standard deviation (SD)] of the mean absolute and relative bias of all paired measurements [27].

To assess the clinical impact of using the Auto40 instead of the FACSCalibur in Chad setting, the sensitivity and the specificity of the Auto40 was calculated to identify patients who had with the FACSCalibur a CD4 T cell count below 200 cells/μl, the threshold of immune-restoration under ART and the threshold for therapeutic initiation according to the 2006-revised WHO recommendations (WHO, 2007), 350 cells/μl, the new threshold for ART initiation for adults and children aged more than 5 years according to the 2010-revised WHO guidelines [2], or 750 cells/μl and%CD4+ ≤25%, the new absolute and percent CD4 T cell count WHO thresholds for ART initiation in children aged between 24 and 59 months [2]. For clinical significance of the measurement differences on treatment decision, the Cohen’s *k* coefficient was calculated on study population [29].

## Results

### Accuracy of direct volumetric CD4 T cell measurements by the Auto40 flow cytometer in Chad

A total of 258 EDTA-blood samples with correct pre-analytical preparation were obtained from 182 HIV-1-infected adults (median age, 33.3 years; range, 18–59, 60 males), and 76 HIV-1-infected children older than 5 years (median age, 6.2 years; range, 5–17, 35 males). The majority of study patients (74%) were taking ART according to the WHO recommendations for resource-limited settings, whereas 26% of them were naïve for ART. No pregnant woman was included in the study.

**Figure 2:**
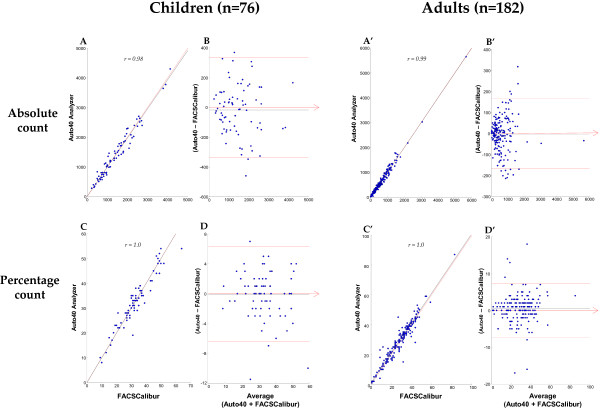
**Results from CD4 T cell count measurements in 182 HIV-1-infected adults and 76 HIV-1-infected children older than 5 years, expressed in absolute number (cells/μl) and in percentage carried out in parallel by the Auto40 flow cytometer and by the FACSCalibur. A**, **A**’, **C** and **C**’. Passing-Bablok agreement tests between the CD4 T cell count results obtained by Auto40 and FACSCalibur flow cytometers, in absolute number (**A** and **A’**) and in percentage (**C** and **C’**). The diagonal dotted line represents the ideal line (no bias). The full line represents the regression line of the distribution; **B**, **B**’, **D** and **D**’. Bland-Altman analyses on the relative differences between the CD4 T cell counts obtained by Auto40 and FACSCalibur flow cytometers compared with the average CD4 T cell count, in absolute number (**B** and **B’**) and in percentage (**D** and **D’**). The full line represents the mean relative difference, and the dotted lines represent the superior and inferior limits of agreement. The arrow corresponds to the x abscise axis.

The absolute and relative bias (and the limits of agreement) for CD4 T cell counting in absolute count and percentage are shown in Table 1 for the study in adults and children, by the Apogee Auto40 flow cytometer and the FACSCalibur, and in Table 2 at various CD4 T cell count ranges. In addition, the Figure 2 depicts the Passing-Bablok agreement test and the Bland-Altman analyses between the CD4 T cell count results obtained by Auto40 and FACSCalibur flow cytometers, in absolute number and in percentage, in the 182 study HIV-1-infected adults and 76 HIV-1-infected children older than 5 years.

**Table 1:** CD4 T cell counting in absolute cells/μl and percentage in 182 HIV-1-infected adults and 76 HIV-1-infected children older than 5 years, by the Apogee Auto40 flow cytometer at the hôpital Militaire D’Instruction, N’Djamena, Chad, and by the FACSCalibur at the hôpital de la Liberté, N’Djamena

**Categories**		**Adults**	**Children**
**Number**		**N = 182**	**N = 76**
**Absolute CD4 T cells (cells/μl)**			
Apogee Auto40	Mean ± SD	627 ± 591	1432 ± 821
FACSCalibur	Mean ± SD	635 ± 588	1449 ± 835
Absolute bias (limits of agreement)^a^	Mean (±1.96 SD)	−16.0 (−38.2;6.1)	30.0 (−14.7;88.7)
Relative (%) bias (limits of agreement)^b^	Mean (±1.96 SD)	1.0 (−1.8;3.8)	0.3 (−2.2;2.7)
**Percentage of CD4 T cells (%CD4)**			
Apogee Auto40	Mean ± SD	29.1 ± 12.8	32.1 ± 10.1
FACSCalibur	Mean ± SD	28.5 ± 12.5	32.3 ± 10.6
Absolute bias (limits of agreement)^a^	Mean (±1.96 SD)	−3.0 (−7.1;1.1)	1.0 (−0.4;2.4)
Relative (%) bias (limits of agreement)^b^	Mean (±1.96 SD)	−11.0 (−25.8;3.8)	3.0 (−1.3;5.3)

^a^ The Bland Altman analysis was carried out to calculate the absolute bias and limits of agreement which are the 95% confidence intervals (±1.96 × SD) of the mean bias of all paired measurements in a given category [27];

^b^ The Pollock analysis was carried out to calculate the relative bias and limits of agreement which are the 95% confidence intervals (±1.96 × SD) of the mean bias of all paired measurements in a given category [28].

SD: Standard deviation.

**Table 2:** CD4 T cell counting in absolute count and percentage in 258 HIV-1-infected individuals, by the Apogee Auto40 flow cytometer at the hôpital Militaire D’Instruction, N’Djamena, Chad, and by the FACSCalibur at the hôpital de la Liberté, N’Djamena, at various CD4 T cell count ranges according to the FACSCalibur results

**Categories**		**< 200 cells/μl**	**200 – 350 cells/μl**	**> 350 cells/μl**
**Number**		**N = 27**	**N = 43**	**N = 188**
Absolute CD4 T cells (cells/μl)*				
Apogee Auto40	Mean ± SD	118 ± 161	278 ± 129	2990 ± 3745
FACSCalibur	Mean ± SD	100 ± 135	215 ± 103	3017 ± 3756
Absolute bias (limits of agreement)^a^	Mean (±1.96 SD)	−4.0 (−33.8;+25.8)	10.0 (−70.0;+90.0)	12.0 (−160.7;+184.7)
Relative (%) bias (limits of agreement)^b^	Mean (±1.96 SD)	62.0 (−134.0;+258.0)	7.0 (−27.2;+41.2)	−3.0 (−12.4;+6.4)
Percent CD4 T cells (%CD4)**				
Apogee Auto40	Mean ± SD	10.0 ± 12.7	19.5 ± 6.3	55.1 ± 44.5
FACSCalibur	Mean ± SD	9.0 ± 11.3	18.0 ± 4.2	53.3 ± 41.7
Absolute bias (limits of agreement)^a^	Mean (±1.96 SD)	1.0 (−1.8;+3.8)	2.0 (−2.3;+6.3)	2.0 (−3.4;+7.4)
Relative (%) bias (limits of agreement)^b^	Mean (±1.96 SD)	6.0 (−10.3;+22.3)	7.0 (−13.2;+27.2)	2.0 (−4.6;+8.6)

* Results in absolute CD4 T cell counts by Auto40 and FACSCalibur were correlated using the non-parametric Passing–Bablok regression analysis (< 200 cells/μl: r = 0.86, slope = 1.11, intercept = −12.0; 200 – 350 cells/μl: r = 0.68, slope = 1.31, intercept = −96.5; > 350 cells/μl: r = 0.98, slope = 1.02, intercept = −24.8);

** Results in percentage CD4 T cell counts by Auto40 and FACSCalibur were correlated using the non-parametric Passing–Bablok regression analysis (< 200 cells/μl: r = 0.89, slope = 1.00, intercept = 0.0; 200 – 350 cells/μl: r = 0.89, slope = 1.00, intercept = 0.0; > 350 cells/μl: r = 0.95, slope = 1.00, intercept = 0.0);

^a^ The Bland Altman analysis was carried out to calculate the absolute bias and limits of agreement which are the 95% confidence intervals (±1.96 × SD) of the mean bias of all paired measurements in a given category [27];

^b^ The Pollock analysis was carried out to calculate the relative bias and limits of agreement which are the 95% confidence intervals (±1.96 × SD) of the mean bias of all paired measurements in a given category [28].

SD: Standard deviation.

Mean ± SD of CD4 T cells/μl expressed in absolute number was 864 ± 761 cells/μl (range, 5–5639) by Auto40 flow cytometer, and 975 ± 765 cells/μl (range, 5–5674) by FACSCalibur (P > 0.5). The non-parametric Passing-Bablok regression analysis on all 258 available T cell results expressed in absolute count revealed a high correlation between CD4 T cell counts obtained by Auto40 flow cytometer and FACSCalibur (r = 0.99) with a slope of 0.98 (95% IC: 0.96 – 1.01) and an intercept of +4.4 (95% CI: -18.0 – 26.8). The mean absolute bias measured by Bland-Altman analysis, and the mean relative bias measured by Pollock analysis, between CD4 T cells/μl obtained by Apogee Auto40 flow cytometer and FACSCalibur over the entire range of CD4 T cell results, were −9.4 cells/μl (95% CI: -23.5 – 4.7) with limits of agreement from −15 to 93 cells/μl, and +2.0% with limits of agreement from −0.9 to 4.9%, respectively.

Mean ± SD CD4 T cell count in percentage was 30.1 ± 12.1%CD4+ (range, 1–87) by Auto40 flow cytometer, and 29.6 ± 12.1%CD4+ (range, 1–83) by FACSCalibur (P > 0.5). Results of CD4 T cell count in percentage by Auto40 flow cytometer and FACSCalibur were highly correlated by regression analysis (r = 0.96) with a slope of 0.96 (95% IC: 0.92 – 0.99) and an intercept of +1.7 (95% CI: 0.6 – 2.9). The mean of absolute bias and relative bias between percentage of CD4 T cells obtained by Auto40 flow cytometer and FACSCalibur were +0.4%CD4 (95% CI: -0.02 – 0.86) with limits of agreement from −2.4 to 0.3%CD4, and +3.0% with limits of agreement from −6.6 to 0.6%, respectively.

The number of outliers showed a trend to be higher when the CD4 T cell count was expressed in absolute count (16/258 = 6.2%) than when it was given in percentage (10/258 = 3.9%), although the difference was not statistically significant (P = 0.22 by Student *t* test).

### Sensitivity and specificity to identify clinically-relevant thresholds by the Auto40 flow cytometer

The sensitivity and specificity of CD4 T cell counting by the Auto40 flow cytometer to identify the patients with less or more than 200 CD4 T cells/μl, 350 CD4 T cells/μl, 750 CD4 T cells/μl and 25%CD4+, were calculated on the 258 available CD4 T cell count measurements, and are depicted in the Table 3. Whatever the thresholds considered, the concordance between the Auto40 flow cytometer and FACSCalibur methods was high, and the decision did not differ between study blood samples according to both methods (κ = 0.96 to 0.98; P < 0.01).

**Table 3:** The sensitivity and specificity of CD4 T cell counting by the Auto40 to identify patients having less than (or more than) 200 CD4 T cells/μl, 350 CD4 T cells/μl, 750 CD4 T cells/μl and 25%CD4+, calculated on the 258 available CD4 T cell count measurements

		**Sensitivity**	**Specificity**	**Cohen’s κ coefficient**^$^
	200 CD4 T cells/μl*	89%	99%	0.97
**Thresholds**	350 CD4 T cells/μl**	94%	98%	0.98
	750 CD4 T cells/μl***	99%	96%	0.97
	**25%CD4**	94%	98%	0.96

* 200 CD4 T cells/μl: Threshold of immune-restoration under antiretroviral treatment and the threshold for therapeutic initiation according to the 2006-revised WHO recommendations [30];

** 350 CD4 T cells/μl: New WHO threshold for antiretroviral treatment initiation in adults and children aged more than 5 years [3];

*** 750 CD4 T cells/μl and 25%CD4+: WHO thresholds for antiretroviral treatment initiation in children aged between 24 and 59 months [2];

^$^A 10% bilateral range (*i.e.*, counts between 190 and 210 CD4 T cells/μl for the threshold at 200 CD4 T cells/μl; counts between 332 and 367 CD4 T cells/μl for the threshold at 350 CD4 T cells/μl; counts between 712 and 787 CD4 T cells/μl for the threshold at 750 CD4 T cells/μl; and counts 23.7 and 26.2%CD4+ for the threshold at 25%CD4+) were considered similar.

## Discussion

In the present study, we carried out a new evaluation of the biological performances of the Auto40 flow cytometer in Chad focusing on the HIV-infected adult and children populations, independently of the manufacturer, and in complement to those previously reported in Senegal [15] and Cameroon [19]. Indeed, several evaluations by at least 3 independent laboratories in the field are strongly recommended for effective validation of the newly introduced affordable CD4 T cell measurement technologies to be used in resources-constrained settings [6,21]. Our results confirm that the Auto40 flow cytometer, whether operated in the field in Chad by a laboratory technician, has an acceptable performance compared with the FACSCalibur for CD4 T cell quantification expressed in absolute number as well as in percentage in HIV-infected adult and children patients. The Auto40 flow cytometer results in absolute number and percentage which gave high correlations with those obtained by the reference flow cytometer method. The correlation was maintained at different CD4 T cell count ranges over all the dynamic range of values in absolute number (until 5639 CD4 T cells/μl) as well as in percentage (until 83%CD4+). CD4 T cell counting by CD45-assisted Auto40 allowed to identify the majority of adults with CD4 T cells below 200 cells/μl or 350 cells/μl, and of children below 750 cells/μl or 25%CD4+, demonstrating the capacity of Auto40 to accurately assess the major thresholds in absolute number (200 cells/μl, 350 cells/μl and 750 cells/μl) or in percentage (25%CD4+), used in clinical practice to initiate or follow ART in adults and children. Taken together, these findings demonstrate that the simplified, single-platform, volumetric, CD45-assisted pan-leucogating Auto40 flow cytometer is a reliable alternative flow cytometer for CD4 T lymphocyte enumeration to be used in routine for immunological monitoring according to the 2010-revised WHO recommendations in HIV-infected adults as well as children living in resource-constrained settings.

Our study confirms and extends the interest of the Auto40 analyzer for CD4 T cell enumeration in developing countries, as recently reported in Senegal by Dieye et al. [15], and in Cameroon by Mbopi-Kéou et al. [20]. The Senegalese validation used the initial Auto40 version based on primary CD4 gating and focused exclusively on CD4 T cell counting in absolute number [15]. However, as the percentage of CD4 T cells is essential for monitoring HIV disease progression in children less than 5 years [2,14], the primary CD4 gating format of the Auto40 analyzer that only measure absolute CD4 T cell numbers, was being modified by the manufacturer in 2006 to measure the percentage CD4 T cells. Thus, our observations extent the previous study in Senegal, by demonstrating that the updated version of the Auto40 flow cytometer, now based on a pan-leucogating protocol using anti-CD45 and anti-CD4 monoclonal antibodies, is not only valid for CD4 T cell enumeration in absolute number, but also in percentage. Finally, the present validation of the Auto40 analyzer in Chad reinforces and improves the previous Senegalese and Cameroonese conclusions on the clinical interest of the Auto40 flow cytometer for immunological monitoring of HIV-infected adult and children. The present study however was conducted under controlled conditions in a reference laboratory and by qualified technicians. Further studies are needed to confirm the reliability of the Auto40 analyzer for routine usage in the field.

Immunophenotyping by flow cytometry is the reference method for the enumeration of the CD4 marker on T lymphocytes [31]. Initially, the use of large panel of reagents and stringent gating techniques for full lymphocyte subset analysis has been shown to be superfluous and expensive [32]. The simplified flow cytometric pan-leucogating protocol is based on a sequential strategy by initially gating on the total white blood cell population (CD45-positive population), which then serves as the common denominator to enumerate CD4-positive T lymphocytes instead of primarily gating on the error prone lymphocyte population [33]. The specific use of the side scatter parameter allows for the discrimination of monocytes due to the high side scatter and low CD4 expression of monocytes thereby enabling accurate CD4-positive T cell enumeration. The CD4-positive lymphocytes are low side scatter and high CD4 expressing cells [34]. The pan-leucogating method has been demonstrated to allow accurate CD4 T cell counting, when used in dual platforms [34-36] as well as single-platforms, either volumetric [37] or bead-based [38,39].

The performance of single-platform, volumetric flow cytometric systems operating with CD45-based gating and generic monoclonal antibodies has been previously reported for a limited number of commercially available analyzers, including mainly the microcapillary-based Guava AutoCD4/CD4%™ (Merck Millipore LS International Hayward, CA, USA) [24] and the CyFlow SL_3™ (Partec GmbH, Munster, Germany) [40]. Both latter analyzers have proven their usefulness for validated absolute and percentage of CD4 T cell counting in the field [24,40]. These single-platforms using only a flow cytometer and volumetric systems allow counting CD4 T cells in a fixed volume, thus leading to a considerable reduction in the costs of CD4-positive T cell enumeration by comparison to microbead-based systems [31]. In addition, the compact low-range single-platforms Auto40 and CyFlow SL_3™ are portable desk-top flow cytometers that does not require optical alignment, can run on a 12-volt car battery, and can be connected to a laptop computer, suggesting their possible use in health care mobile unit that could make CD4 T cell enumeration available in remote or hard-to-reach locations, as previously shown for the Auto40 flow cytometer installed in health mobile unit in Cameroon [20]. These observations raise the issue of decentralization of immunological monitoring in large areas where laboratories are remote, using health mobile units capable of performing screening and immunological monitoring of HIV-infected patients [20,41].

One of the additional main features of the Auto40 analyzer is the use of stabilized monoclonal antibodies. The assay reagents can be stored for prolonged period of time (up to 12 months) at high temperature without any loss of biological activity [42]. The use of thermo-resistant reagents allowed reliable measurements of CD4 T cell count, both in absolute and percentage terms, under unfavourable conditions like high temperatures in the tropics, as in remote areas where the conditions met for the storage of reagents (cold chain), particularly during shipment and delivery, is not guaranteed [43]. The thermo-stable monoclonal antibodies used in the present study can be kept for as long as one year at room temperature, *i.e.* 30°C, and has been chosen for all laboratories equipped by an Auto40 flow cytometer in Chad. The possibility of long-term storage of reagents at room temperature for one year should facilitate the planning of laboratory activities and reduce the costs related to loss of reagents stability. Overall, the use of thermo-stable reagents increases the accessibility to flow cytometry testing. However, while the manufacturing costs related to the antibody stabilization procedure does not exceed 15% of the original cost [42], the final sale prices of thermo-resistant reagents marketed by Inodia (France) is higher than 40% compared to those of liquid reagents.

In the present study, all specimens used for the evaluation were fresh and not travelled. This practice represents the current situation in Chad. Indeed, there is no organization of blood samples transportation thorough the country, mainly because of heavy logistical constrained in a very large country, which is furthermore affected by a hard tropical climate. In contrast, the so-called “Conseil National de Lutte contre le SIDA”, N’Djamena, Chad decided to decentralize the CD4 analyzers, and acquired from 2003 to 2012 around twenty CD4 analyzers to be installed in laboratory facilities thorough the country.

The accurate determination of CD4 T cells is of crucial clinical importance for caring adults or children infected with HIV or suffering from AIDS. Although classical flow cytometry represents the reference method for CD4 T cell enumeration, the feasibility of flow cytometric methods in the field remains controversial in resource-poor settings [8]. In the Cameroonese experience, well-trained technicians were able to use the Auto40 flow cytometer with low intra- and inter- run precisions ranging from 4.8% to 5.5% and from 5.3% to 7.9%, respectively, thus less than 10% [19,20], considered as acceptable for routine clinical practice [15], and comparable to other published reports using single-platform flow cytometers [10,24,40,44]. The low observed variability of the Auto40 flow cytometer confirmed that single-platform flow cytometric methods are more reproducible than dual-platform [45]. Higher precisions for high than for low CD4 T cell count have been previously reported with single-platform volumetric flow cytometric method [40]. Finally, the Auto40 system showed a high capacity of accurately discriminate CD4 T cell values around the relevant CD4 T cells thresholds recommended by the 2010-revised WHO guidelines to initiate and follow ART in HIV-infected adults or children, as previously reported [15,19,46].

## Conclusions

In conclusions, the Auto40 flow cytometer constitutes a promising system for performing single-platform absolute and percent CD4 T lymphocyte counts with excellent reproducibility, and should facilitate wider access to CD4 T cell enumeration for adults and children infected with HIV infection living in resource-constrained countries.

## Competing interests

The authors declare that they have no competing interests.

## Authors’ contributions

DK, ANM and LB conceived and designed the research, and analyzed the results, performed the statistical analyses and drafted the manuscript with MAJ; DK and NN have performed the experiments; BD was directly involved in the implementation of Auto40 analyzers in Chad; NDO discussed the medical validity of CD4 T counting by the Auto40 analyzer. All authors read and approved the final manuscript.
